# A cost benefit analysis of a virtual overdose monitoring service/mobile overdose response service: the national overdose response service

**DOI:** 10.1186/s13011-023-00565-8

**Published:** 2023-10-04

**Authors:** William Rioux, Benjamin Enns, Jennifer Jackson, Hena Quereshi, Mike Irvine, S. Monty Ghosh

**Affiliations:** 1 Department of Medicine, Faculty of Medicine & Dentistry, University of Alberta, Edmonton, AB Canada; 2 Centre for Health Evaluation and Outcome Sciences, Vancouver, BC Canada; 3 Faculty of Nursing, University of Calgary, Calgary, AB Canada; 4 British Columbia Centre for Disease Control (BCCDC), Vancouver, BC Canada; 5 Department of Internal Medicine, Faculty of Medicine & Dentistry, University of Alberta, Edmonton, AB Canada

**Keywords:** Mobile overdose response services, Opioids, Overdose, Drug poisoning, Cost analysis, Emergency services

## Abstract

**Background:**

The overdose crisis continues across Canada which calls for novel harm reduction strategies. Previous research indicates that a majority of eHealth solutions are cost-effective however current literature on the cost-benefit of eHealth for harm reduction is sparse. The National Overdose Response Service (NORS) is a Canada-wide telephone-based harm reduction service. Service users can call the phone number and connect to a peer who can virtually monitor the substance use session and dispatch appropriate interventions in the case of overdose.

**Objectives of the research/project:**

We aim to assess the cost-benefit of NORS by comparing the estimated cost-savings from prevented overdose mortality to the operating costs of the program, alongside healthcare costs associated with its operation.

**Methods:**

Data around systems costs and operational costs were gathered for our calculations. Our primary outcome was cost-benefit ratios, derived from estimates and models of mortality rates in current literature and value of life lost. We presented our main results across a range of values for costs and the probability of death following an unwitnessed overdose. These values were utilized to calculate cost-benefit ratios and value per dollar spent on service provision by NORS over the length of the program’s operation (December 2020–2022).

**Results:**

Over the total funded lifespan of the program, and using a Monte Carlo estimate, the benefit-to-cost ratio of the NORS program was 8.59 (1.53–15.28) per dollar spent, depending on estimated mortality rates following unwitnessed overdose and program operation costs. Further, we conservatively estimate that early community-based naloxone intervention results in healthcare system savings of $4470.82 per overdose response.

**Conclusions:**

We found the NORS program to have a positive benefit-to-cost ratio when the probability of death following an unwitnessed overdose was greater than 5%. NORS and potentially other virtual overdose monitoring services have the potential to be cost-effective solutions for managing the drug poisoning crisis.

**Supplementary Information:**

The online version contains supplementary material available at 10.1186/s13011-023-00565-8.

## Introduction

The drug poisoning epidemic has seen higher mortality rates than those seen from Covid-19 in some Canadian provinces [1–3]. In addition to the human toll, there are significant economic consequences of these drug poisonings impacting demand for healthcare services. In 2017, substance use cost Canadians an estimated $46.0 billion, led to more than 275,000 hospitalizations, and contributed to the loss of 75,000 lives [4]. In the U.S. this cost is estimated to be 22 times higher at $1.02 trillion annually attributed to tangible costs like healthcare and productivity as well as intangible costs including loss in quality of life [5].

Thus far, supervised consumption services (SCS) also known as safe injection sites (SIF)s have been studied as an efficient and cost-effective option for curbing the harms due to the unregulated drug poisoning epidemic [6, 7]. Virtual overdose monitoring services (VOMS), more recently also known as Mobile Overdose Response Service (MORS), are a novel harm reduction strategy that aims to bolster SCS reach and has become formalized over the course of the pandemic. MORS/VOMS aims to address those who use drugs alone in private residences, who represent a majority of drug poisoning deaths according to 2022 coroners statistics from the Canadian province of British Columbia [8]. These services generally include two different methodologies to monitor a substance use session: (1) Connection to a live operator (usually a person with lived or living experience of substance use or “peer”), or (2) A timer-based application that requires individuals to refresh a countdown, these services and their recent evidence have been summarized in recent scoping reviews [9, 10].

Currently, available MORS/VOMS in Canada include the National Overdose Response Service (NORS) [11] and or app-based services including the BeSafe Brave app [12], Connect by Lifeguard [13], and the Digital Overdose Response Service [14] with others available in other countries including Never Use Alone (NUA) in the United States [9]. The primary objective of MORS/VOMS are to prevent overdose-related mortality through early intervention of drug poisonings with secondary objectives of connecting individuals to appropriate healthcare service [11, 12, 15]. Recent pilot studies indicate that MORS/VOMS are an effective addition to current harm reduction services, extending the geographic reach and peer connection provided by supervised consumption sites [16, 17]. NORS is a Canadian telehealth/hotline service in which users are connected to peer service employees (staff members with lived and living experience of substance use) through an automated call linkage system, staff will stay on the line during the substance use session and activate a predetermined emergency response plan should it be required [11].

While the efficacy and cost-effectiveness/benefit of SCS is well supported in the literature [6, 7], to our knowledge there have been no cost-benefit evaluations of MORS/VOMS. Many of the current harm reduction services result in reductions in mortality, HIV, and other infectious diseases, but have limited reach due to their geographic location [18]. Previous qualitative research indicates that MORS/VOMS provides an option to those who do not feel comfortable or cannot access these services [19, 20]. Due to the virtual nature of these services, however, some of the integral supports and savings associated with SCS may not be available such as the reduction in harms associated with preventing sexually transmitted and blood-borne illnesses. Evaluating the cost-effectiveness and cost-benefit of MORS/VOMS presents challenges wherein clients of these services are under no obligation to provide their name or healthcare information to retain client anonymity thus we are unable to directly link service usage to healthcare data.

In this study, we aimed to compare the value of lives saved by Canada's NORS to the operational and response costs required to deliver the intervention [11]. We examine the population-level benefit of NORS and the findings from our analysis may be used to inform future funding allocation on behalf of political decision-makers and assess the long-term feasibility of these services. Of note, this cost-benefit analysis is only one evaluation piece to a broader cost-effectiveness evaluation and budget impact analysis currently being established for NORS.

## Methods

### Description of intervention

NORS users are connected to peer operators who gather basic information about the individual and their location to be able to enact an emergency response in the event of an overdose. Peer operators additionally call clients after an overdose event to ensure their wellness and, to date, no deaths have occurred on the line. The NORS line has been operational since December 2020 as a volunteer service but only began receiving funding from Health Canada in April 2021. For our analysis, only program data was extracted directly from operator-imputed call logs during the years in which NORS was funded. Where possible call-takers ascribe unique identifiers to clients based on gathered information in the form of a caller code which allows for tracking of unique clients over time. Accurate states of overdoses addressed per month were captured, as well as response type (Emergency Medical Services (EMS) vs. community-based response). Gender, age, and other demographic data were not necessarily recorded within the call logs throughout the intervention and were omitted from our analysis.

### Study design

Our cost-benefit analysis plan was developed from a previous study examining the economic impact of physical SCSs [7]. We examined a number of key metrics and values including the operational cost specifically of NORS, the number of overdose deaths prevented, costs of healthcare services provided, and the economic value of deaths prevented [6, 7]. Our primary outcome measure was net benefit and benefit-to-cost ratios generated by the NORS program, using the economic value of deaths prevented, compared to the operational costs. Base case calculations were performed in Excel, and additional Monte Carlo estimations and sensitivity analyses were conducted in R. The Checklist for Health Economic Evaluation Reporting Standards was utilized to help guide the reporting of this study [21].

### Costs and health outcomes

As each province in Canada has different associated healthcare costs, efforts were made to utilize average costs per jurisdiction. A payer’s perspective was utilized to examine the cost impact of the service. The payer in this study refers to the federal government which had provided funding to run this program as well as the provincial government which provides health systems-based funding such as hospital costs, and EMS call-outs. Discussions with NORS operators, Health Service providers, and people of lived experience were conducted to determine which key aspects of MORS/VOMS and outcome metrics were required for the evaluation.

The key metric of analysis for this study was remote overdose management, as defined as virtually or remotely identifying someone as being unrousable after using an illicit substance while using MORS/VOMS. Identification of an overdose results in medical interventions wherein MORS/VOMS operators contact either EMS services or a community-based support system to administer naloxone and reverse the overdose. Ethics was obtained from the University of Calgary (REB22-0735).

### Analysis plan

Our analysis takes the form of two separate but interrelated valuations which only look to examine the value of the NORS program. We examine the cost-benefit of NORS by both varying costs of service operation and value generated by the service across diverse rates of fatal unwitnessed overdose which are seen in recent literature.

### Estimation of costs

Two categories of system-related costs were examined while holding the value generated from the service which consisted of: (1) operating costs for providing the NORS service, and (2) cost of ambulance, emergency department support, and possible admission for individuals who had become unresponsive on the phone and required additional intervention and support.

### Operating costs at NORS (Oc)

The first component of our analysis looked at the operating cost of NORS per call. This includes wages for the operators, administrators, evaluation team, and technology-related costs. These are considered fixed costs associated with the service up to a point in which incremental service utilization would require further proportional support thereby becoming a variable cost. As NORS is a newly established service, they had not reached a point in which proportional support was required.

### Systems cost of medical services for overdoses

The second component of our analysis looked at the cost of medical services for clients after a drug poisoning/overdose event. These costs include the clients’ journey from pre-hospital ambulance dispatch and also initial emergency department evaluation. They also consider potential hospital stays. The following formula was utilized:

$$\begin{array}{c} \text{TMSc} = Cost\ of\ \mathrm{T}_{\text{OD}} + Cost\ of\ \mathrm{T}_{\text{FC}} + Cost\ of\ \mathrm{T}_{\text{CR}}\\ \text{OR}\\ \text{TMSc} = \{(\mathrm{T}_{\text{EMS}})(\$518)\}+\{(\mathrm{T}_{\text{EMS}})(\$1061 + \$196.83 + \$125)\} +\\ (\mathrm{T}_{\text{EMS}})(0.33)(\$7803) + (\mathrm{T}_{\text{FC}})(\$518)+ (\mathrm{T}_{\text{CR}})(\$125) \end{array}$$

Where:

- TMSc is the total cost of medical services including emergency and medical supports per year in 2022 CAD.
- T_EMS_ is the total number of overdoses per year responded to by EMS.
- T_FC_ is the number of false positive calls per year.
- T_CR_ is the number of community responses per year as opposed to emergency call outs.
- T_OD_ is the number of overdoses taken to hospital.

TMSc is the total cost of emergency and medical support per year. This cost is mitigated with community-based responses in which a community member instead of EMS services responds to the client and reverses a potential overdose. T_OD_ is the total number of clients who overdosed and were taken to emergency services per year. The value was built from ambulance fees, the cost of ED visits, evaluation by a physician, and naloxone administration. Each province has a unique ambulance fee. A national average of $518 (range $240–848) [22–25] was chosen as representative of national costs. Costs of emergency department visit for overdose in Alberta is $1061 as per the latest cost evaluation by the Canadian Institute of Health Information [26]. This value is based on the comprehensive ambulatory classification system and selected codes based on previous studies [6]. The cost of physician assessment was $176 [6] but adjusted to $196.83 to account for inflation. While intramuscular naloxone is the most abundant and utilized form of naloxone and is the cheapest at $30 a kit, many responders throughout Canada utilize the more expensive intranasal naloxone at a cost of $125 a device [27]. We opted to be conservative and utilize the value of an intranasal naloxone kit exclusively.

Based on our data and post-drug poisoning callbacks, no individual was admitted to intensive care, although several were admitted for short hospital stays. Average rates of hospitalization from overdoses were estimated to be 33% (range 26-40%) [28]. The average national hospitalization cost of overdoses should the individual be admitted was $7803(range $6,620 − 13,647) [29]. All results were reported in 2022 CAD in Table 1 with inflation adjustments for costs listed in previous years.

**Table 1:** Cost-benefit analysis parameter inputs including reference cost and range

Table 1	Reference cost	Reference
Emergency Medical Services	$518.00 (240–848)	[22–25]
Emergency Visit	$1,061.00	[26]
Physician Assessment	$176.00	[6]
Nasal Naloxone	$125.00	[27]
Hospitalization	$7803($6,620 − 13,647)	[29]
NORS operational cost	$1,366,749.00	Appendix 1

### Estimating the value of life

The primary benefit of this service is preventing death and long-term health complications among people who use substances (PWUS) experiencing an overdose. Due to the aforementioned challenges in data linkage through the collection of public health numbers, we were only able to estimate the value of life lost due to drug poisoning. There is a general lack of data around an exact average and median age of death from opioid poisonings, with most epidemiological reports in Canada providing age ranges between 30 and 39 [3, 30]. We utilized Alberta data to determine the median age of death from drug toxicities which was 38 [30]. This is in line with age range data provided by various provinces throughout the country [3].Given the lack of overall effectiveness data, and examining previous cost-benefit studies of supervised consumption sites, we decided to utilize previously used harm reduction evaluation methodologies to calculate the value of life lost to society [7], we apply the average Canadian income in our relevant age group btween 35 and 44 years of age. This value is $73,530 2022 CAD [31]. As a conservative approach, based on literature and expert opinion we assumed 67% would be gainfully employed [32]. This addition to the average age of retirement of 64.8 [33] would result in a potential loss of 26.8 productive years of life. Thus, when only looking at the value of human life, one life lost to overdose may result in $1,320,304.68 lost to Canadian society.

As identified in previous studies [6, 7], the utilization of productive life lost is controversial, and indeed some argue that there may be little loss of productivity or lost wages from PWUS, or that death from substance poisoning may actually save the system money, but this raises key ethical issues. From an ethical standpoint, it is problematic to value one individual’s life above another. To estimate the economic value of a prevented death, and as has been done previously with these types of studies, we looked at the potential value lost to society from a life lost to a drug overdose/poisoning using average incomes. This was deemed the most tangible way to conduct this evaluation given the lack of data in this field, but was also in line with previous studies examining this concern. This also provides the benefit of allowing us to compare our analysis with existing literature on physical supervised consumption services that have been evaluated. To date no studies have examined the employment and health and social service utilization demographics of MORS/VOMS users and as such we have not factored any of these metrics into our evaluation, although there are estimates of employment rates with PWUS.

An additional note is that individuals who survive a drug poisoning/overdose event have often been considered as high health system users. Of note, this typically pertains to individuals who experience homelessness or concomitant mental health disorders and other extreme vulnerabilities [34]. The intervention provides harm reduction access to a different demographic of PWUS, especially individuals who are using indoors in housed facilities who are not necessarily congruent with high systems users. For this reason, we have not factored this into estimating the current and future benefits of the intervention.

### Survival following an unwitnessed or unattended overdose

In order to further understand and comprehend drug poisoning outcomes, we looked at survival rates to examine the counterfactual response, where we would determine how many individuals would have survived had no intervention been provided either via EMS or community-based response. The probability of death resulting from an unwitnessed overdose is unknown and difficult to estimate. Previous modeling and expert consensus studies have estimated this value to range from 8 to 80% [35–37]. We adopted the most recent and arguably most thorough value reported by Irvine et al. (2019) of 10% which refers to the mortality rate of an overdose without naloxone administration to report our base-case results. This is likely a conservative estimate as mortality rates have more than doubled in Canada since the data for this study was collected in 2016 [3]. Additionally, this study was examining the utility of take-home naloxone in particular, and that this probability was an estimate of mortality of unwitnessed overdose not specifically resuscitated with take-home naloxone kits. As such the probability of mortality is higher. The larger value of 80% was based on expert consensus from a previous study using a modified-Delphi methodology examining the transition patterns of individuals who use oral opioids to illicit opioids, as well as patterns and availability of naloxone in PWUS. Part of the analysis examined estimated probabilities of death following an unwitnessed overdose, accounting for the increase in toxic drug supply from adulterants such as carfentanil and benzodiazepines [37]. This estimate is likely an overestimate of the actual mortality rate and serves as our ceiling estimate. In order to better understand the uncertainty around the variability of mortality from an unwitnessed overdose, a Monte Carlo simulation was conducted using NORS data, which provided an estimate of 30 deaths averted or a mortality rate of 45%.

In order to better assess survival from an unwitnessed or unattended overdose we utilized the following formula:

$${\mathrm N}_{\mathrm p}=\mathrm N\left({\mathrm P}_{\mathrm n}-{\mathrm P}_{\mathrm i}\right)$$

Where:

- N_P_ = Number of overdoses prevented.
- N = Number of unique individuals who used the line i.e. utilized the intervention.
- P_n_ = Probability of death from an unwitnessed overdose or unattended overdose based on our Monte Carlo simulation which is 45% of individuals who did not use the intervention.
- P_i_ = Probability of death from using the hotline or any other virtual supervised consumption service i.e. Individuals who used the intervention.

### Cost-benefit analysis and outcomes

#### Total benefit and net savings

In order to determine total benefit and net savings, we took the difference between the total value of deaths avoided subtracted by the total operational costs of VOMS and the total costs of medical services provided.

$${\mathrm T}_{\mathrm{CB}}:\:\mathrm{Estimated}\;\mathrm{value}\;\mathrm{of}\;\mathrm{deaths}\;\mathrm{averted}-{\mathrm{TO}}_{\mathrm C}-{\mathrm{TM}}_{\mathrm{SC}}$$

#### Community-based response savings

In order to determine the net value of community-based cost savings, where the overdose respondent is not a professional emergency response service, we conducted a cost difference analysis. The following formula was utilized:

$$\mathrm{Cost}\;\mathrm{Difference}\:=\:\mathrm{Number}\;\mathrm{of}\;\mathrm{Community}\;\mathrm{Based}\;\mathrm{Overdoses}\;(\mathrm{Medical}\;\mathrm{cost}\;\mathrm{per}\;\mathrm{overdose}\;\mathrm{responded}\;\mathrm{to}\;\mathrm{by}\;\mathrm{EMS}\;-\;\mathrm{Cost}\;\mathrm{of}\;\mathrm a\;\mathrm{community}\;\mathrm{response})$$

### Sensitivity analysis

In order to account for the variability in key model parameters, we present our main results in a two-way sensitivity analysis across variable program costs and mortality rates without intervention as described in recent literature. We additionally calculate the value and benefit of increasing costs of program funding as current values reflect skeleton costs and increased operational funding will likely be required.

## Results

### Survival following an unwitnessed or unattended overdose

Within our evaluation, we noted that there were 23 unique individuals (with an additional 4 individuals who were unknown) who had an overdose event and no deaths (Pi = 0), however, if the sample size were to increase, we would begin to see a rise in this Pi value. In order to predict the future impacts of this intervention, and others like it if it were scaled, a sensitivity analysis was conducted examining the 95% CI of Pi, assuming the number of deaths follows a Poisson distribution. This sensitivity analysis is demonstrated in Table 2.

**Table 2:** Sensitivity analysis of the number of deaths averted using the intervention based on Monte Carlo simulation estimates

Number of Unique Individuals who use NORS and had an overdose event.	10,000	4500	4490	4480	4470	4460	4450	4440	4430
	9000	4050	4041	4032	4023	4014	4005	3996	3987
	8000	3600	3592	3584	3576	3568	3560	3552	3544
	7000	3150	3143	3136	3129	3122	3115	3108	3101
	6000	2700	2694	2688	2682	2676	2670	2664	2658
	5000	2250	2245	2240	2235	2230	2225	2220	2215
	4000	1800	1796	1792	1788	1784	1780	1776	1772
	3000	1350	1347	1344	1341	1338	1335	1332	1329
	2000	900	898	896	894	892	890	888	886
	1000	450	449	448	447	446	445	444	443
Probability of death following an unwitnessed overdose (in percentage)		0	0.1	0.2	0.3	0.4	0.5	0.6	0.7

### Estimated costs and health outcome data from the NORS program

Since the start of NORS’ funding in April 2021, the cost of service fluctuated per year depending on the number of EMS call outs that occurred and service utilization (Table 3), while the overall operational costs remained static. From the 11 times in which a community response was initiated, the healthcare system saved a calculated $47,858.93 2022 CAD or $4,350.81 per overdose response.

**Table 3:** NORS Outcome data including overdoses and service utilization

	Funded 1	Funded 2	
	Apr 21 - Mar 22	Apr 22 Dec 22	Total
Total number of service calls	3512	1647	5159
Total number of substance use phone calls	2023	1090	3113
Total number of mental health phone calls	969	391	1360
Total of referrals to other services	41	79	120
Total Overdoses	45	15	60
Overdose in unknown individuals	2	2	4
Overdose in unique individuals	13	10	23
Unique + Unknown	15	12	27
Total emergency medical call outs for overdoses	36	13	49
Total community-based overdose responses	9	2	11
Emergency Medical Service call outs for non-overdose emergencies^*^	3	1	4
False positive Emergency Medical Service call outs for assumed emergencies	2	0	2

^*^Non-overdose emergencies include acute psychosis, domestic violence, and property break-ins during a substance use session

 Additionally Table 4 examines the cost per life saved in the program as calculated from the estimated number of overdose deaths prevented per years using our Monte Carlo estimation.

**Table 4:** Cost per life saved and total cost of prevented overdose deaths associated with NORS

	April 2021- March 2022	April 2022- December 2022	Total
Estimated number of overdose deaths prevented per year based on our Monte Carlo simulation:	21	7	28
*Total cost of prevented overdose deaths from NORS using using value of one life lost* ($1,320,304.68)	$27,726,388.30	$9,242,132.76	$36,968,521.10
*Total Number of life years saved*:	562.8	187.6	750.4
*Overall Cost per life year saved from using NORS*	$49,265.08	$49,265.10	$49,265.09

### Calculated costs

To date, the NORS program has utilized $1,366,749 CAD in operational funding. A percentage breakdown of costs associated with the program is presented in Appendix 1. Furthermore, the calculated medical system costs associated with the program are provided in Table 5.

**Table 5:** Direct healthcare system costs associated with NORS

Time Period	Value of lives saved based on estimated mortality rate Using Monte Carlo estimate (0.45) and Range (0.08–0.8) CAD 2022	Operating Costs CAD 2022	Medical Costs CAD 2022	Net Benefit (Range)	Cost/Benefit ratio with Monte Carlo estimate and Range
April 2021- March 2022	$7,723,782.38 ($1,373,116.86 - $13,731,178.67)	$787,500.00	$163,290.52	$6,772,991.86 ($422,326.34 -$12,780,388.15)	8.12 (1.44–14.44)
April 2022- December 2022	$5,941,371.06 ($1,056,243.74 - $10,562,437.44)	$580,875.00	$58,435.66	$5,302,060.40 (416,933.08- 9,923,126.78)	9.29 (1.65–16.52)
Total	$13,665,153.40 ($2,429,360.61 - $24,293,606.11)	$1,368,375.00	$221,726.18	$12,075,052.26 ($839,259.43- $22,703,504.93)	8.59 (1.53–15.28)

### Estimated benefits

Estimated cost of lives lost if NORS did not exist: December 2020 there were 66 overdose events recorded, 27 were from unique callers and 4 from unknown callers. In order to more accurately represent the benefits of the program, we only looked at values during which the program was funded in which there were 23 unique overdoses and 4 overdoses from unknown callers. We again opt to use the more conservative value of 23 unique client overdoses. Due to the aforementioned challenges in estimating the probability of mortality for unwitnessed overdose, we present the values of the service across the mortality present in the current literature which can be seen in Table 6.

**Table 6:** Outlines the program costs on the Y axis as a percentage of overall costs. 100% delineated the current program costs. The X-axis describes the mortality rate from unwitnessed overdose seen across current literature. Restults are presented as the net benefits of the program with the cost-benefit ratio in brackets

*Net Benefits of the NORS program in 2022 CAD (value per dollar spent)*											
*Costs of NORS program as a percentage of current operational budget.*	*200*	*- $ 529,115.57)* *(0.82)*	*$ 78,224.58* *(1.03)*	*$ 3,114,925.35* *(2.05)*	*$ 6,151,626.11* *(3.08)*	*$ 9,188,326.88* *(4.11)*	*$ 10,706,677.26* *(4.62)*	*$ 12,225,027.64* *(5.13)*	*$ 15,261,728.40* *(6.16)*	*$ 18,298,429.17* *(7.19)*	*$ 21,335,129.93* *(8.21)*
	*180*	*- $ 255,440.57* *(0.90)*	*$ 351,899.58* *(1.13)*	*$ 3,388,600.35* *(2.26)*	*$ 6,425,301.11* *(3.39)*	*$ 9,462,001.88* *(4.52)*	***$ 10,980,352.26*** ***(5.09)***	*$ 12,498,702.64* *(5.66)*	*$ 15,535,403.40* *(6.79)*	*$ 18,572,104.17* *(7.92)*	*$ 21,608,804.93* *(9.05)*
	*160*	*$ 18,234.43* *(1.01)*	*$ 625,574.58* *(1.26)*	*$ 3,662,275.35* *(2.54)*	*$ 6,698,976.11* *(3.78)*	*$ 9,735,676.88* *(5.04)*	***$ 11,254,027.26*** ***(5.67)***	*$ 12,772,377.64* *(6.30)*	*$ 15,809,078.40* *(7.56)*	*$ 18,845,779.17* *(8.82)*	*$ 21,882,479.93* *(10.08)*
	*140*	*$ 291,909.43* *(1.14)*	*$ 899,249.58* *(1.42)*	*$ 3,935,950.35* *(2.84)*	*$ 6,972,651.11* *(4.26)*	*$ 10,009,351.88* *(5.68)*	***$ 11,527,702.26*** ***(6.39)***	*$ 13,046,052.64* *(7.10)*	*$ 16,082,753.40* *(8.52)*	*$ 19,119,454.17* *(9.94)*	*$ 22,156,154.93* *(11.37)*
	*120*	*$ 565,584.43* *(1.30)*	*$ 1,172,924.58* *(1.63)*	*$ 4,209,625.35* *(3.26)*	*$ 7,246,326.11* *(4.89)*	*$ 10,283,026.88* *(6.52)*	***$ 11,801,377.26*** ***(7.33)***	*$ 13,319,727.64* *(8.15)*	*$ 16,356,428.40* *(9.78)*	*$ 19,393,129.17* *(11.41)*	*$ 22,429,829.93* *(13.03)*
	100*	***$ 839,259.43*** ***(1.53)***	***$ 1,446,599.58*** ***(1.91)***	***$ 4,483,300.35*** ***(3.82)***	***$ 7,520,001.11*** ***(5.73)***	***$ 10,556,701.88*** ***(7.64)***	***$ 12,075,052.26*** ***(8.59)***	***$ 13,593,402.64*** ***(9.55)***	***$ 16,630,103.40*** ***(11.46)***	***$ 19,666,804.17*** ***(13.37)***	***$ 22,703,504.93*** ***(15.28)***
	*80*	*$ 1,112,934.43* *(1.85)*	*$ 1,720,274.58* *(2.31)*	*$ 4,756,975.35* *(4.61)*	*$ 7,793,676.11* *(6.92)*	*$ 10,830,376.88* *(9.23)*	***$ 12,348,727.26*** ***(10.38)***	*$ 13,867,077.64* *(11.53)*	*$ 16,903,778.40* *(13.84)*	*$ 19,940,479.17* *(16.15)*	*$ 22,977,179.93* *(18.45)*
	*60*	*$ 1,386,609.43* *(2.33)*	*$ 1,993,949.58* *(2.91)*	*$ 5,030,650.35* *(5.82)*	*$ 8,067,351.11* *(8.74)*	*$ 11,104,051.88* *(11.65)*	***$ 12,622,402.26*** ***(13.10)***	*$ 14,140,752.64* *(14.56)*	*$ 17,177,453.40* *(17.47)*	*$ 20,214,154.17* *(20.39)*	*$ 23,250,854.93* *(23.30)*
	*40*	*$ 1,660,284.43* *(3.16)*	*$ 2,267,624.58* *(3.16)*	*$ 5,304,325.35* *(7.90)*	*$ 8,341,026.11* *(11.85)*	*$ 11,377,726.88* *(15.79)*	***$ 12,896,077.26*** ***(17.77)***	*$ 14,414,427.64* *(19.74)*	*$ 17,451,128.40* *(23.69)*	*$ 20,487,829.17* *(27.64)*	*$ 23,524,529.93* *(31.59)*
		*8%*	*10%*	*20%*	*30%*	*40%*	45% **	*50%*	*60%*	*70%*	*80%*

*Overdose mortality rate without intervention*

*Demarcates the current operational costs of NORS,

** Demarcates the estimated mortality rate from an unwitnessed overdose from out Monte Carlo simulations

### Cost-benefit results

Overall our findings indicate that above a 5% rate of mortality for unwitnessed overdose, the NORS program provided positive cost-benefit. The actual cost-benefit ratio range, utilizing our conservative and ceiling mortality estimates, were between between 1.53 and 15.28, with our Monte Carlo probability based estimate being 8.59.

## Discussion

The focus of this study was the cost-benefit of the NORS program in Canada. The economic value estimated from NORS was substantial, offsetting the costs associated with the operation of the service, with benefit-to-cost ratios ranging from 1.53 to 15.28, depending on changes in operational costs and the probability of death following an unwitnessed overdose. Estimates for the rate of mortality following an unwitnessed overdose vary widely across literature, a previous article by Irvine et al. (2021) provides a modeled value of the rate of death from unwitnessed overdose in British Columbia. at 10%. This value is likely underestimated the current mortality rate from an unwitnessed overdose in British Columbia, noting that it includes values predominantly from the pre-fentanyl era. Additionally, mortality rates have nearly doubled in British Columbia since the time of data collection [38]. Despite utilizing the most conservative mortality rate statistics present in literature, the program continues to be cost-effective and provides value for dollar. It is also believed that scaling of the service and engaging more unique individuals would likely yield higher cost-to-benefit ratios which could be witnessed as the service grows.

Uniquely Table 2 demonstrates the potential impact of scaling NORS and other MOR services such as Connect by Lifeguard, Brave, Never Use Alone, and other automated services in regards to overdose and deaths prevented using the intervention. It also notes that the intervention is by far not perfect, with an incremental increase in the risk of death even using the intervention utilizing a Poisson distribution.

A substantial amount of cost savings occurred from community-based responses as opposed to EMS call-outs, where individuals near the person who overdosed would be contacted instead of EMS to respond to a suspected overdose. False positive call-outs for overdoses were fairly minimal and did not impact costs amounting to a total system cost of $1,036 in 2022 CAD. Of note, the cost-benefit ratio varied depending on the number of unique callers who overdosed. This phenomenon is similar to physical supervised consumption sites where individuals overdose on multiple occasions while using the service wherein previous studies note that the top 1% of individuals account for 25% of overdose events [39].

While we focused exclusively on dollars saved from overdoses, which was the primary focus of these services, there could be additional impacts and cost savings from NORS which were not considered. For instance, NORS provides peer-based mental health support as well as substance-induced psychosis de-escalation which helps prevent potential transfers to the hospital as well as other wellness benefits. NORS also provided referrals for clients to other services including community harm reduction services, social services such as income support, drug treatment programs, and clinics that provide opioid agonist treatment, all of which could add additional benefit. Furthermore, a majority of the studies which looked to determine the benefits of SCSs primarily derived their benefits from the prevention of sexually transmitted and blood-borne illnesses. While NORS does distribute naloxone kits and needles via mail, this is not a core function of its service. To our knowledge, most MORS/VOMS do not distribute these either and this may be an additional opportunity to be able to decrease the harms associated with the use of illicit substances. One previous implementation of this methodology was Philadelphia’s mail-order naloxone program which was demonstrated to be a viable strategy for increasing access to harm-reduction supplies [40].

It should be noted that while our data specifically applies to NORS, the methodology, calculations, and formula is applicable to digital phone and mobile app-based programs such Connect by Lifeguard, the Brave App, and DORS by taking the number of unique lives saved then subtracting the manufacturing and operational cost of the various smartphone applications, as well as the cost of emergency call outs, false positive calls, and community-based responses from them.

It is also important to note that other similar programs such as Never Use Alone [41] in the United States operate without funding. As seen within our results, these services provide both a large return on investment and reduce the harms associated with illicit drug use. While the Never Use Alone data is outside of the scope of this analysis, the healthcare cost savings provided by this program likely provides a large benefit to society. Given the responsibility around monitoring for drug poisonings and the impact of these programs, increased advocacy in regard to funding with government and donors to help formalize and spread these services is warranted. Furthermore, increased uptake of these services would likely result in an increase in the cost-benefit of these interventions. As a result, novel strategies to promote these services among people who use drugs such as the use of messaging on naloxone kits [42] or other public health dissemination strategies should be utilized to extend the reach of these harm-reduction services. An enhanced economic evaluation looking at improvements in quality of life, a more in-depth analysis of EMS call-outs, hospital admissions, social service referrals, and disease/presentation severity scores for hospital evaluations, could help further enhance the findings of this study.

Lastly, while MORS/VOMS present some advantages in reducing harm reduction service barriers (i.e.: geographic access, stigma, routes of administration, and creating gender-responsive care environments [15, 20] they currently do not support all individuals who use drugs. Statistics indicate that less than half of individuals accessing SCS [43] have access to a phone, in contrast, however, in a study of 421 homeless adults moving into permanent supportive housing, 94% possessed a cell phone [44]. Future studies should focus on the potential impacts of narrowing the digital divide for these populations and their resultant healthcare access. In lieu of these solutions, continued funding and support should be provided to previously researched harm reduction programs including SCS, needle, and syringe programs.

### Limitations

When interpreting our findings, it is important to note a few key limitations. To begin, as the service is nationwide, with each province having unique service costs, attempts were made to use the national average costs of services. It was difficult to estimate lost years of productivity given the lack of general data around the percentage of vulnerable PWUS who work and have housing, salary by age category, maternal leave, etc., and as such we may have overestimated this value. We have been conservative in our other calculations however which would account for some of this overestimation. Second, our study used population-level data collected from NORS call logs, these make it difficult to draw firm conclusions on the healthcare costs associated with each individual. Future research could look to link service users and healthcare records. Second, while we evaluated the costs of mortality from overdose, we did not take into consideration morbidity. Of note, all overdoses on the line were followed up with and there were no admissions to intensive care units or concerns with hypoxic brain injury warranting long-standing admission or physical rehabilitation. If this was the case, the cost of medical services would considerably increase, and the overall benefits would decrease. Rates of hospitalization following non-fatal overdose range between 26 and 40% [28], furthermore rates of severe injury after non-fatal overdose which are associated with high health service utilization and mortality burden averaged 5% [45]. While there have yet to be any serious adverse events as confirmed with follow up calls, due to the small sample size of the population studied these outcomes may result in the future and would significantly impact the costs-benefit ratio. Fourth, our analysis assumes that all overdoses which activated emergency response on NORS would have otherwise been unwitnessed. Lastly, while all overdose was tracked and logged, there was some missing data in regard to client names and identification. While call takers attempted to use unique identifiers for callers, not all of these were logged.

## Conclusion

We found the NORS program to have a positive benefit-to-cost ratio when the probability of death following an unwitnessed overdose was greater than 5%, with the benefit-to-cost ratio increasing to 1.53 per dollar spent when the probability of death was 10% and to 8.59 when the probability of death was 45% based on our Monte Carlo simulation. NORS and other MORS/VOMS may be a cost-effective solution for addressing substance-based overdose mortality in communities without access to physical supervised consumption sites, and to those who use drugs alone.

### Supplementary Information

**Additional file 1.**

## Data Availability

The data that support the findings of this study are available on request from the corresponding author, M.G. The data are not publicly available due to the sensitivity of substance use and interview transcripts containing information that could compromise the privacy of research participants.

## Abbreviations

MORS: Mobile Overdose Response Services
SCS: Supervised Consumption Services
NORS: National Overdose Response Service
DORS: Digital Overdose Response Service
EMS: Emergency Medical Services
