# Supplementary material for: A cost benefit analysis of a virtual overdose monitoring service/mobile overdose response service: the national overdose response service
Source: Subst Abuse Treat Prev Policy. 2023 Oct 4;18:57. doi: 10.1186/s13011-023-00565-8 (PMC10548617; doi:10.1186/s13011-023-00565-8)
Supplement: Supplementary file 1 — Additional file 1. [file 13011_2023_565_MOESM1_ESM.docx]

| **BUDGET (EXPENDITURE-BASED)** | | | | |
| --- | --- | --- | --- | --- |
| **Org. Name:** | **Grenfell Ministries Inc** | | | |
| **Initiative Title:** | **National Overdose Response Service (NORS)** | | | |
| **Initiatives can be a minimum of one year in duration and a maximum of five years. The recommended duration is two to three years. Please only use the budget years that are associated with your proposed activities.** |  |  |  | Percentage Breakdown |
| Operational Manager (1 FTE) | $70,000 | $70,000 | **$140,000** | 9% |
| Employer's Share of Payroll Deductions (Benefits) | $17,500 | $17,500 | **$35,000** | 2% |
| Supervisors (8 FTE) | $390,000 | $390,000 | **$780,000** | 50% |
| Employer's Share of Payroll Deductions (Benefits) | $66,300 | $66,300 | **$132,600** | 8% |
| Program Evaluator (0.5 FTE) | $40,950 | $40,950 | **$81,900** | 5% |
| Employer's Share of Payroll Deductions (Benefits) | $6,962 | $6,962 | **$13,923** | 1% |
| **GOODS & SERVICES (CONTRACTORS)** |  |  |  |  |
| Legal Advisors | $20,000 | $20,000 | **$40,000** | 3% |
| Volunteer Training | $8,000 | $8,000 | **$16,000** | 1% |
|  |  |  |  |  |
| **TRAVEL & ACCOMMODATIONS** |  |  |  | 0% |
| Transportation | $4,500 | $4,500 | **$9,000** | 1% |
| Accommodation | $1,200 | $1,200 | **$2,400** | 0% |
| Meals & Incidentals | $480 | $480 | **$960** | 0% |
| **GOODS & SERVICES (MEETINGS/EVENTS)** |  |  |  |  |
| Room/Space Rental | $0 | $0 | **$0** | 0% |
| Hospitality | $0 | $0 | **$0** | 0% |
| Services: translation | $29,000 | $29,000 | **$58,000** | 4% |
| Services: Marketing/PR/Printing etc | $100,000 | $100,000 | **$200,000** | 13% |
| **MATERIALS & SUPPLIES** |  |  |  |  |
| Office Supplies: computers | $15,000 | $0 | **$15,000** | 1% |
| Teleconference services | $1,140 | $1,140 | **$2,280** | 0% |
| Printing / Dissemination | $468 | $468 | **$936** | 0% |
| Postage | $3,400 | $3,400 | **$6,800** | 0% |
| **EQUIPMENT** |  |  |  |  |
| Office Equipment | $3,400 | $3,400 | **$6,800** | 0% |
| Other: Expert community advisory group | $2,000 | $2,000 | **$4,000** | 0% |
| Other: Participant Honoraria | $6,000 | $6,000 | **$12,000** | 1% |
| Other: Focus group | $1,200 | $1,200 | **$2,400** | 0% |
| **RENT & UTILITIES** |  |  |  |  |
| Rent | $0 | $0 | **$0** | 0% |
| Utilities | $0 | $0 | **$0** | 0% |
| **Sub-total** | **$0** | **$0** | **$0** | 0% |
| **KNOWLEDGE TRANSLATION & DISSEMINATION** |  |  |  |  |
| Peer driven knowledge dissemination | $0 | $2,000 | **$2,000** | 0% |
| **PERFORMANCE MEASUREMENT & EVALUATION** |  |  |  | 0% |
| Performance Measurement / Evaluation | $0 | $0 | **$0** | 0% |
| **Sub-total** | **$0** | **$0** | **$0** | 0% |
| **Other (please specify):** |  |  |  |  |
| Other | $0 | $0 | **$0** | 0% |
| **Sub-total** | **$0** | **$0** | **$0** | 0% |
| **Total** | **$787,500** | **$774,500** | **$1,561,999** |  |
| **Total spent** |  | **$580,875** | **$1,368,375** |  |
|  |  |  |  |  |
